# Designing the multimedia system for improving promotion of college students’ psychological capital

**DOI:** 10.1016/j.heliyon.2024.e25362

**Published:** 2024-02-01

**Authors:** Jinghui Zeng, Yangfen Chen, Yingying Zheng

**Affiliations:** a College of Business Administration, Zhejiang Technical Institute of Economics, Hangzhou, China; b The Affiliated School of the Future Science and Technology City to Hangzhou Normal University, Hangzhou, China; c School of Physical Education and Health, Wenzhou University, Wenzhou, 325035, China

**Keywords:** Multimedia system, College students, Psychological capital, Promotion, College student community

## Abstract

Given the rising psychological challenges encountered by university students, there is an imperative to address the pressing need for enhancing their psychological capital. This study is to design an innovative multimedia system that seeks to offer comprehensive psychological support and promotion mechanisms for university students. This is achieved through the integrated use of various media forms. Multimedia system group counseling was employed to assess and enhance the psychological capital of college students. This study comprises two main components: first, an analysis of the application of multimedia technology in education, and second, an empirical investigation into college students’ psychological capital through a questionnaire survey. The findings reveal that the introduction of group counseling via a multimedia system significantly enhances the psychological capital of college students. This improvement in psychological capital positively impacts the well-being and mental states of students and contributes novel ideas to mental health education for college students. The effectiveness of the group counseling intervention scheme within the multimedia system is evident, suggesting its potential for widespread adoption. The utilization of multimedia systems in educational settings emphasizes the importance of positive psychology for students and contributes to cultivating a positive and healthy psychological state. This study serves as a valuable reference for enhancing the psychological capital of college students, focusing on aspects such as independent thinking, decision-making, and execution.

## 1 Introduction

College students constitute a distinctive demographic characterized by mature physiological development yet often exhibit diverse and evolving psychological states. These states are influenced by a myriad of factors, including varying social environments, family backgrounds, and individual personalities [1]. In the context of independent life and self-study, college students, due to their limited social experience and relatively fragile resilience, are susceptible to various challenges. If these challenges are not appropriately addressed, they may lead to psychological distress and disorders. Recognizing the significance of mental health issues among college students, society and educational institutions, particularly colleges and universities, have increasingly focused on mental health education initiatives. These initiatives encompass the introduction of psychological capital concepts, emphasis on positive psychology, and the cultivation of independent thinking, decision-making, and execution capabilities [2]. In the course of addressing mental health concerns, multimedia technology has emerged as a transformative tool. By integrating multimedia elements such as audio, videos, animation, graphics, and texts into teaching materials, the conventional classroom teaching paradigm has undergone substantial changes [3]. Learning content is no longer confined to traditional tools like chalk and blackboard; instead, it is presented in a diverse and visualized manner, significantly enhancing learner engagement and improving the efficiency of content delivery. The advent of multimedia technology opens up new avenues for enhancing the psychological capital of college students. Nevertheless, empirical studies on the intervention and development of college students’ psychological capital remain scarce. This gap in research fails to meet the evolving needs of college students in strengthening their competitive advantages and aligning higher education with future developmental trends [4].

Psychological capital encompasses a positive psychological state during the stages of growth and development, encompassing elements such as self-efficacy, optimism, resilience, and hope [5]. However, the existing body of research on the psychological capital of college students is limited. Given the frequent occurrence of campus crises, there is a call to prioritize research exploring the positive dimensions of human psychological capital. Such investigations can offer a comprehensive perspective for mental health education initiatives tailored to college students [6]. The challenges inherent in university life can have a profound impact on the mental well-being and academic performance of students. Despite this, there is limited research exploring innovative approaches to enhance the psychological capital of university students. Notably, Chen et al. (2023) [7] underscored the importance of psychological capital, encompassing self-efficacy, optimism, hope, and resilience, as a means to mitigate challenges and foster positive outcomes. In a cross-sectional study conducted by Joseph et al. (2023) [8] involving 212 management students in Kerala, India, a multi-stage random sampling method was employed for data collection. The findings reveal a significant influence of psychological capital on the happiness of management students. Both perceived employability and psychological capital exhibit a positive correlation with life satisfaction. Additionally, perceived employability acts as an intermediary factor in the relationship between psychological capital and life satisfaction.

Recognizing the proven efficacy of multimedia interventions in positively shaping psychological factors, this study endeavors to craft a bespoke multimedia system. This system is meticulously designed to bridge existing research gaps, with the overarching objective of fortifying the psychological capital of university students. Anticipated as innovative research, the endeavor is poised to contribute novel theoretical and practical perspectives, thereby laying the groundwork for deeper exploration within the domain of mental health among university students.

Hence, this study endeavors to investigate the interplay between college students’ attitudes toward seeking psychological help, their psychological capital, and their mental health. This exploration will be conducted through the administration of a psychological capital questionnaire tailored for college students. The central research question guiding this study is: Does the multimedia system exert a significant influence on augmenting the psychological capital of university students? Grounded in this inquiry, we posit the following research hypotheses.

Hypothesis 1 The utilization of the multimedia system leads to a significant increase in the self-efficacy levels of university students.

Hypothesis 2 The multimedia system exerts a positive influence on the hope levels of university students, cultivating more optimistic expectations for the future.

Hypothesis 3 Active participation in the multimedia system significantly enhances the optimistic attitudes of university students.

Hypothesis 4 The levels of psychological capital among female students are anticipated to be significantly higher than those among male students.

Across various dimensions of psychological capital, female students are anticipated to showcase heightened self-efficacy, foster more positive expectations, and demonstrate superior adaptability. In contrast to their male counterparts, female students are expected to recover more swiftly from adversity through positive communication. Ultimately, a meticulous comparison between the experimental group and the control group, coupled with a thorough analysis of relevant data, substantiates these hypotheses, contributing to a comprehensive understanding of the application of multimedia systems in augmenting the psychological capital of university students. This study aims to furnish mental health institutions in colleges and universities with a robust theoretical foundation for preventing and addressing mental health issues among college students. The ultimate goal is to facilitate the holistic development of their physical and mental well-being.

## 2 Literature review

Numerous scholars in both China and foreign countries have conducted extensive research on the challenges and opportunities presented by multimedia technology in psychological education. Reference [9] proposes that, in light of the challenging employment landscape, college students should bolster their employability and acquire professional knowledge. The study emphasizes the close relationship between the improvement of college students' employability and the effective development of their psychological capital. Consequently, there is an inevitable demand for research on psychological capital dimensions such as hope, optimism, resilience, emotional intelligence, and self-efficacy, which significantly contribute to college students' employability. Reference [10] contends that psychological capital constitutes a key element of enterprises' core competitiveness, serving as a crucial indicator for talent recruitment, selection, and training assessments. Improving college students' psychological capital, according to this perspective, plays a pivotal role in enhancing their employability. The exploration of psychological capital extends into the field of college students' human resources development. The establishment of a micro-intervention mechanism focusing on the dimensions of self-efficacy, optimism, hope, and resilience aims to cultivate positive energy and unlock potential, thereby elevating students' competitiveness in the job market. Reference [11] studies the relationship among college students' psychological capital, overall well-being, and mobile phone dependence is examined, providing a theoretical foundation for addressing mobile phone dependence issues. Conducting a questionnaire survey on 1000 students from a university in Shanxi Province, the research reveals that college students exhibit a significant dependence on mobile phones. While this dependence varies across academic performance, it shows no notable gender differences. The study establishes a significant correlation between college students’ mobile phone dependence, overall well-being, and psychological capital. Moreover, it identifies that overall happiness and psychological capital serve as significant predictors of mobile phone dependence [12].

The analysis of the psychological capital structure above reveals notable variations in the perspectives of different researchers. This diversity arises due to the relative immaturity of theoretical research on psychological capital and the exploration of the subject from diverse perspectives and backgrounds [13]. Despite these differences, there is a degree of consensus among researchers regarding the fundamental structure of psychological capital. This study aligns with the prevailing research consensus and adopts a four-dimensional structure, encompassing self-efficacy, hope, optimism, and resilience. The focus of this investigation extends to the realization and application of a multimedia teaching system. This includes considerations such as the configuration of multimedia classroom hardware equipment, the design of a teaching system based on computer or network technology, the performance testing of the system, and the establishment of a multimedia teaching platform.

## 3 Methods

### 3.1 Application of multimedia technology in higher education

Multimedia technology encompasses the use of computers to integrate and process text, graphics, images, sound, animations, and videos, establishing logical relations and facilitating human-computer interactions [14]. This technology comprises both hardware and software components. Hardware technology encompasses storage, compression, chip, and database technologies, while software technology includes multimedia software development and application. The defining characteristics of multimedia technology include interactivity, integration, real-time functionality, multidimensionalization, and digitization, with interactivity being its key feature [15]. This implies that users can interact with various computer information media, providing a more effective means of controlling and utilizing information. Integration involves the comprehensive processing of diverse information media centered on computers, encompassing both the integration of information media and the integration of the equipment processing these media. Multidimensionalization refers to the diversification of computer-processed media information, while digitization signifies the existence of media in digital form. Real-time functionality denotes the dynamic changes over time in sound and dynamic images (video). Consequently, multimedia technology finds widespread application across various fields, subtly transforming people's lives [16]. For college students, multisensory stimulation proves more effective than multiple sensory stimulations or single sensory stimulation.

When considering the objectives and content, it is advisable to prioritize the size and development efficiency of courseware [17]. Multimedia information technology has the capacity to alter the size, distance, and speed of objects, thereby overcoming the limitations of time and space. Utilizing the multimedia system to enhance college students' psychological capital requires a comprehensive understanding of the system. Given its close association with network content and communication modes, particular attention should be directed towards guiding communication content and mode to effectively leverage multimedia as a tool for enhancing college students' psychological capital [18]. The multimedia system can be employed for group counseling activities aimed at improving college students’ psychological abilities. Additionally, emphasis should be placed on constructing a human-computer interaction environment [19].

### 3.2 Evaluation of students’ psychological capital based on positive psychology

Towards the close of the 20th century, a shift in focus from negative to positive psychology emerged among Western psychologists. This transition led to an emphasis on positive organizational behavior, characterized by positive orientation, measurability, exploitability, state-based attributes, and performance relevance. Psychological capital, distinct from other positive psychological factors, entered the research landscape relatively recently, and its conceptualization and scope remain subjects of ongoing exploration. Initial discussions about psychological capital surfaced in the realms of economics, investment, and sociology [20].

The buffer effect model posits that psychological capital exerts an indirect influence on pertinent outcome variables. Psychological capital, in this framework, sequentially influences individual subjective satisfaction, motivation, and job search behavior [21]. The moderating effect model posits that psychological capital influences relevant outcome variables through a moderating effect. A new research model is proposed, indicating a mutual influence between psychological capital and related outcome variables. The dynamic effect model suggests that psychological capital and related outcome variables interact with each other.

Self-efficacy within the framework of psychological capital encompasses five critical dimensions.

(1) Domain Relevance: Self-efficacy is inherently domain-specific, with confidence confined to a particular domain without easy transferability to others, regardless of familiarity.
(2) Foundation in Proficiency: Proficiency serves as the cornerstone of self-efficacy, with individuals expressing more confidence in tasks characterized by familiarity and experience [22].
(3) Dynamic Nature: Self-efficacy is dynamic, allowing for continuous improvement, even in domains where confidence is established. This adaptability accommodates encounters with challenging tasks [23].
(4) Influence of Others' Opinions: Others' opinions wield a considerable impact on self-efficacy. Observing similar individuals successfully completing tasks contributes to the enhancement of confidence [24].
(5) Continuous Evolution of Individual Self-Efficacy: Individual self-efficacy undergoes constant evolution, shaped by personal efforts, knowledge, and resources [25].

Moreover, possessing necessary resources is viewed as another facet of self-efficacy, enabling individuals to assess whether they possess the requisite “tools” for successful accomplishment, thereby further shaping their self-efficacy positively or negatively.

To qualify as an element of psychological capital, five criteria and four characteristics must be met. The five criteria include: (1) having a source; (2) being measurable; (3) being unique; (4) being subject to change; and (5) being positive. The four characteristics encompass: (1) displaying confidence in tackling challenging tasks and striving for success; (2) maintaining an optimistic attitude toward the present and future, attributing events positively; (3) setting goals and proactively adopting flexible measures to achieve them; (4) exhibiting resilience by quickly recovering from adversity or frustration until success is achieved [26].

**Fig. 1:**
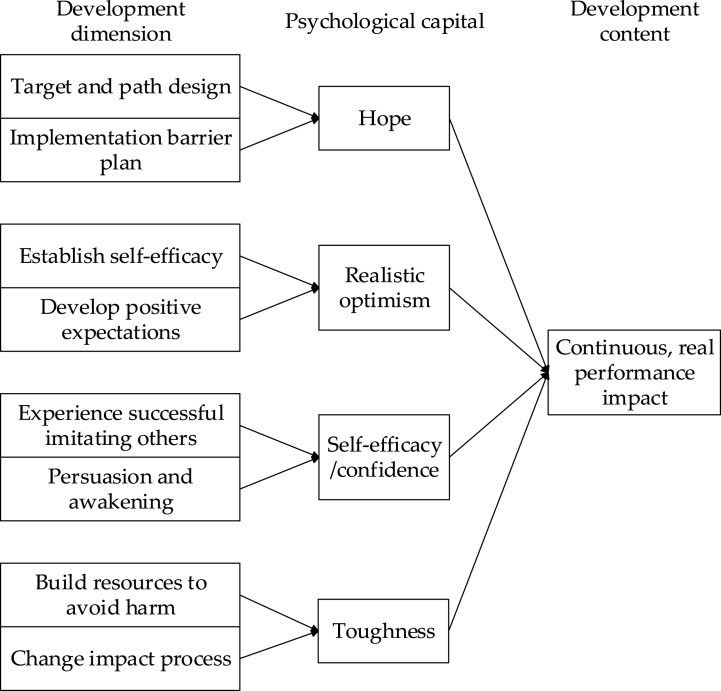
Micro-intervention model of psychological capital.

In accordance with the aforementioned five criteria and four characteristics, individual psychological capital comprises self-efficacy, hope, optimism, and resilience. The micro-intervention model of psychological capital emphasizes the enhancement of hope levels, the cultivation of optimism, the improvement of self-efficacy, and the strengthening of resilience, as illustrated in Fig. 1.

Fig. 1 delineates the fundamental components of the micro-intervention model for psychological capital, encompassing.

(1) Hope: This facet underscores the cultivation and augmentation of individuals' hope levels. This is achieved through the establishment of positive goals and the formulation of pragmatic plans, stimulating proactive behavior.
(2) Realistic Optimism: Individuals are encouraged to maintain a positive and optimistic demeanor while simultaneously fostering a rational awareness of reality. This approach mitigates excessive optimism that may lead to subsequent disappointment.
(3) Self-Efficacy/Confidence: The model focuses on fortifying the sense of self-efficacy, aiming to elevate individuals' confidence levels when confronted with challenges. This, in turn, enhances their capacity to effectively cope with difficulties.

The structure of psychological capital can be categorized into three aspects: state theory, trait theory, and comprehensive theory, as outlined in Table 1.

**Table 1:** List of psychological capital structure.

Three aspects	Measurement dimension
State theory	Self-esteem, self-efficacy, locus of control, emotional stability, extraversion, openness, agreeableness, responsibility, hope state, optimism state, resilience state
Trait theory	Hope, optimism, self-efficacy, resilience, integrity
Comprehensive theory	Hope, optimism, resilience, self-efficacy

### 3.3 Experimental design and hypothesis

The aim of this study is to concentrate on the psychological capital of college students by developing and designing multimedia systems and conducting empirical intervention research. The study seeks to investigate the intervention effect of group counseling based on a multimedia system on college students' psychological capital. Furthermore, the study aims to explore methods to effectively enhance the level of college students' psychological capital, subsequently improving their well-being and mental states, and expanding new perspectives on well-being and mental health education. Through the exploration and design of psychological capital intervention group programs and empirical intervention research, the study aims to identify challenges in improving college students' psychological capital [27]. It is expected that the intervention effect of group counseling based on PCIL theory on college students' psychological capital is investigated, and the methods to effectively improve the level of college students' psychological capital are explored through the group intervention of college students’ psychological capital.

Group intervention for psychological capital proves to be instrumental in elevating the psychological capital of college students, as evidenced by the notable differences observed in the dimensions of psychological capital between the control group and the experimental group post-intervention. The enhancement of college students’ psychological capital is closely linked to a substantial improvement in their well-being and psychological states [28].

For this investigation, students enrolled at Xi'an University of Electronic Science and Technology were selected as the research cohort. Following approvals from both the university authorities and the individual students on November 4, 2022, participants were chosen randomly from the campus pool, specifically targeting those exhibiting lower levels of psychological capital and motivation for engagement. Proactive measures were implemented to augment the credibility and generalizability of the research, including deliberate efforts to expand the sample size. Ultimately, 50 eligible participants were carefully chosen. Following participant selection, half of this cohort (25 students) were randomly assigned to the experimental group, participating in multimedia system group counseling activities spanning eight weeks, from November 5, 2022, to December 24, 2022. Simultaneously, the remaining participants (25 students) were assigned to the control group. Comprehensive participant details are presented in Table 2.

**Table 2:** Questionnaire of undergraduates’ psychological capital group activities.

Classification	Experimental group		Control group	
Gender	Male	Female	Male	Female
Freshman	8	3	6	5
Sophomore	0	5	3	2
Junior	4	2	4	1
Senior	2	1	3	1
Total	14	11	16	9

**Fig. 2:**
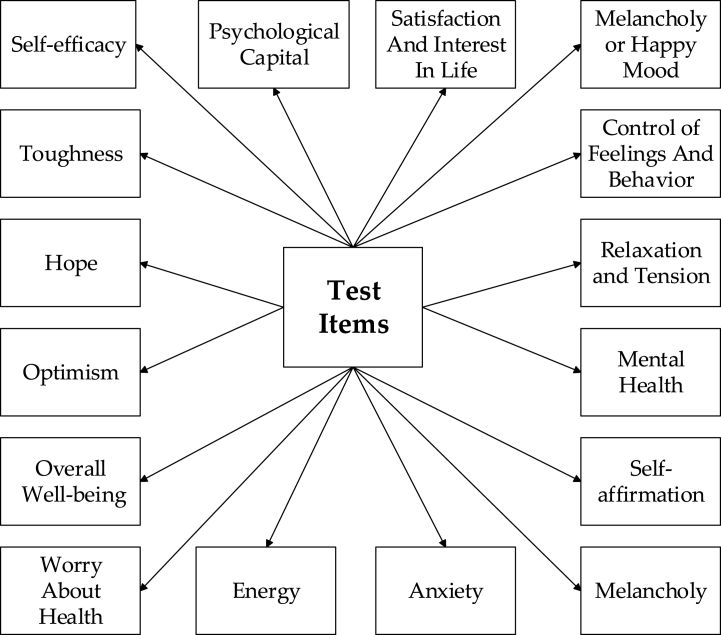
Test items for a comprehensive analysis of psychological capital.

The concept and dimensions of psychological capital are meticulously examined. Based on literature research, observations, communication, and interviews with students, the following hypothesis is posited: the psychological capital of girls surpasses that of boys. Gender differences in personality between boys and girls are noted [29]. In the face of setbacks, boys tend to independently tackle issues, while girls exhibit proficiency in seeking guidance and opinions from others, rendering them more adept at overcoming challenges. The test items are depicted in Fig. 2.

Fig. 2 illustrates the dimensions employed for the comprehensive analysis of psychological capital, encompassing the following key aspects.

1) Self-Efficacy: This dimension measures an individual's confidence in their abilities, gauging their assurance in facing challenges and navigating difficulties.
2) Toughness: This dimension assesses an individual's resilience and capacity to endure adversity and pressure effectively.
3) Psychological Capital: This dimension provides a holistic evaluation of positive mindset, self-efficacy, hope, and coping abilities, reflecting the overall psychological resources of an individual.
4) Satisfaction and Interest in Life: This dimension signifies an individual's overall life satisfaction and interest in various facets of life.
5) Melancholy or Happy Mood: This dimension evaluates an individual's emotional state, determining whether they tend towards a melancholic or happy mood.
6) Control of Feelings and Behavior: This dimension measures an individual's level of self-control over emotions and behavior.
7) Relaxation and Tension: This dimension assesses an individual's degree of relaxation when dealing with stress and tension.
8) Self-Affirmation: This dimension represents an individual's positive self-identification and self-worth.

Through the exploration of these testing dimensions, researchers can attain a comprehensive understanding of the psychological capital status of the participants.

### 3.4 Data acquisition

The methods used in this study primarily encompass literature review, questionnaire surveys, and data analysis. Literature consultation involves an in-depth review of relevant literature in both Chinese and international contexts. Academic literature, books, and papers pertaining to positive psychological capital, well-being, and mental health are scrutinized to extract insights into the connotations, constituent elements, and theoretical frameworks of psychological capital. This literature review lays a robust theoretical foundation for the scientific exploration of college students’ psychological capital and the formulation of the group counseling program. The questionnaire investigation constitutes a crucial aspect of the research methodology, serving as a tool to identify eligible participants for the group counseling program. The questionnaire, designed specifically for college students, undergoes testing across four dimensions: self-efficacy, hope, resilience, and optimism.

The Rickett's five-point scoring method is employed to evaluate responses to the questionnaire. A scale ranging from 1 to 5 is utilized to indicate the level of agreement with each item: “1” refers denoting “totally inconsistent”, “2” for “inconsistent”, “3” for “uncertain”, “4“ for “consistent”, and “5” for “totally consistent.” Notably, for resilience, questions 8, 10, 12, and 14 are subjected to reverse scoring. A higher cumulative score indicates elevated psychological capital in respondents. The correlation between each factor and the total score ranged from 0.67 to 0.73, demonstrating a strong association. Additionally, inter-factor correlations fall within the range of 0.31–0.55, affirming the questionnaire's robust structural validity [30].

**Fig. 3:**
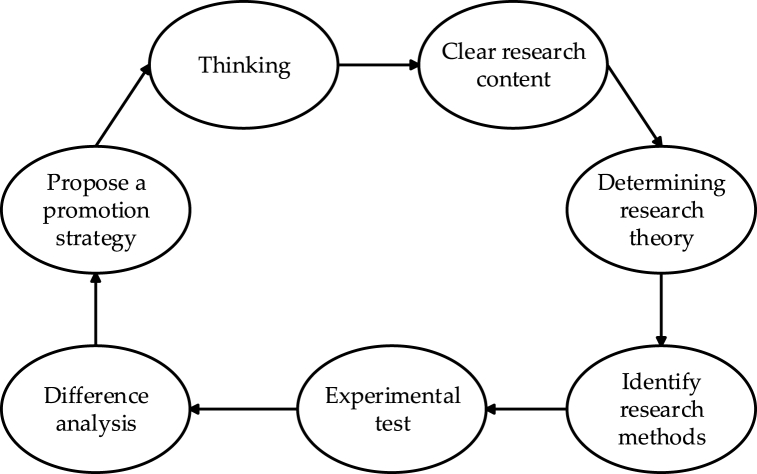
Research route.

In the execution of the group counseling program, a comprehensive assessment of group counseling activities is undertaken through a multi-angle evaluation, utilizing pre-test and post-test scores from both the experimental and control groups. Statistical analysis is performed employing descriptive analysis, the independent sample test, single-factor analysis of variance, and other general analytical methods, along with diverse tests specific to college students' psychological capital. This rigorous analysis aims to furnish data support for a scientifically grounded, systematic, and targeted strategy for enhancing college students' psychological capital. Data analysis and processing are carried out using SPSS 25.0 statistical software. Notably, statistical methods primarily include the independent sample *t*-test and the paired sample *t*-test. The research is characterized by a qualitative and quantitative exploration of college students’ psychological capital, merging theoretical concepts with practical insights. The research route is illustrated in Fig. 3.

Fig. 3 delineates the sequential steps closely associated with the design and implementation of the University Student Psychological Capital Promotion System.

1) Thinking: This initial step involves the preliminary exploration of the University Student Psychological Capital Promotion System concept, contemplating its background, objectives, and the scope of the addressed issues.
2) Clear Research Content: The second step encompasses the determination of the key content of the system, precisely specifying the components and influencing factors of university students' psychological capital.
3) Determining Research Theory: This step involves the selection and clear definition of the theoretical framework supporting the system design, facilitating a better understanding and explanation of its effectiveness.
4) Identify Research Methods: The fourth step revolves around the selection of appropriate research methods to evaluate the implementation effectiveness of the Psychological Capital Promotion System, including data collection and analysis methods.
5) Experimental Test: In this phase, the Psychological Capital Promotion System is implemented, on-site testing is conducted, and practical data regarding the system's effectiveness are systematically collected.
6) Difference Analysis: The sixth step entails a differential analysis of the implementation effects, aiming to gain in-depth insights into the system's performance under varying conditions.
7) Propose a Promotion Strategy: Based on the research results, this final step involves proposing specific strategies to enhance the system, with the ultimate goal of providing more effective support for the psychological well-being of university students.

## 4 Results

An independent sample *t*-test was employed to scrutinize the score differences between the two groups in the positive psychological capital questionnaire, the overall well-being scale, and the general health questionnaire, encompassing each sub-dimension, before the commencement of group counseling. The results are shown in Table 3.

**Table 3:** Analysis of differences in pretests between the control group and experimental group.

Items	Pretest of the control group		Pretest of the experimental group		t	p
	M	SD	M	SD		
Psychological capital	116.1818	13.85509	115.4545	11.97801	0.132	0.8977
Self-efficacy	30.6364	4.12971	30.6364	3.66804	0.000	1.000
Tenacity	26.4545	5.00727	26.1818	5.65364	0.120	0.906
Hope	30.6364	4.63191	28.9091	4.80530	0.858	0.401
Optimism	28.4545	5.04705	29.7273	3.84944	−0.665	0.514
General well-being	68.7273	9.72719	68.7273	12.67352	0.000	1.000
Health concerns	7.8182	2.18258	10.2727	3.40855	−2.011	0.058
Energy	15.6364	3.47197	14.7273	3.16515	0.642	0.528
Satisfaction and interest in life	6.1818	1.07872	5.3636	1.85864	1.263	0.221
Melancholy or happy mood	14.1818	2.60070	13.2727	2.68667	0.806	0.430
Control of feelings and behavior	10.6364	1.28629	10.3636	1.36182	0.483	0.634
Relaxation and tension	14.2727	2.61116	14.7273	4.02718	−0.314	0.757
Mental health	12.8182	3.54452	12.1818	4.44563	0.371	0.714
Self-affirmation	4.0909	2.34327	3.7273	2.90141	0.323	0.750
Melancholy	0.9091	0.70065	0.5455	0.93420	1.033	0.314
Anxiety	1.3636	1.36182	2.0000	1.84391	−0.921	0.369

Note: *p < 0.05; **p < 0.01; ***p < 0.001.

Before the implementation of multimedia system group counseling, both the experimental group and the control group exhibit no significant alterations in the positive psychology questionnaire, general well-being questionnaire, general health questionnaire, and their respective sub-dimensions. Consequently, the members of the experimental and control groups demonstrate homogeneity in terms of psychological capital levels, happiness, and mental health.

The paired sample *t*-test is employed to comprehend alterations in mental capital, happiness, and mental health among participants in their natural state. This analysis scrutinizes score differences in the comprehensive positive mental capital questionnaire, overall happiness scale, general health questionnaire, and other dimensions before and after group counseling. The outcomes are delineated in Table 4.

**Table 4:** Analysis of differences in post-test between the control group and experimental group.

Items	Post-test of the control group		Post-test of the experimental group		t	p
	M	SD	M	SD		
Psychological capital	116.1818	13.85509	117.8182	12.15581	−0.608	0.557
Self-efficacy	30.6364	4.12971	32.0000	3.66060	−1.042	0.322
Tenacity	26.4545	5.00727	26.3636	4.20173	0.069	0.946
Hope	30.6364	4.63191	30.8182	4.55671	−0.091	0.929
Optimism	28.4545	5.04705	28.6364	3.69521	−0.158	0.878
General well-being	68.7273	9.72719	69.2727	7.82420	−0.155	0.880
Health concerns	7.8182	2.18258	8.8182	1.47093	−1.106	0.295
Energy	15.6364	3.47197	16.2727	3.13340	−0.425	0.680
Satisfaction and interest in life	6.1818	1.07872	6.0909	1.64040	0.136	0.894
Melancholy or happy mood	14.1818	2.60070	13.0909	2.02260	1.096	0.299
Control of feelings and behavior	10.6364	1.28629	10.0909	1.04447	0.944	0.367
Relaxation and tension	14.2727	2.61116	14.9091	2.25630	−0.517	0.616
Mental health	12.8182	3.54452	12.7273	4.98179	0.47	0.964
Self-affirmation	4.0909	2.34327	3.8182	3.18804	0.238	0.817
Melancholy	0.9091	0.70065	0.8182	0.98165	0.232	0.821
Anxiety	1.3636	1.36182	1.2727	1.48936	0.142	0.890

Note: *p < 0.05; **p < 0.01; ***p < 0.001.

No significant variations are observed in the scores between the pre-test and post-test phases for the control group in the comprehensive positive psychological capital questionnaire, general well-being questionnaire, and general health questionnaire, including their sub-dimensions. This suggests that the psychological capital, happiness, and mental health of control group members remained consistent before and after the group counseling activities.

Furthermore, paired sample t-tests are conducted to assess the impact of group counseling on the psychological capital, happiness, and mental health of the experimental group. These tests compare the scores of experimental group members in the total positive psychological capital questionnaire, overall happiness scale, and general health questionnaire, as well as various dimensions, before and after group counseling. The findings are presented in Table 5.

**Table 5:** Analysis of differences between pretest and post-test in the experimental group.

Items	Pretest of the experimental group		Post-test of the experimental group		t	p
	M	SD	M	SD		
Psychological capital	115.4545	11.97801	140.2727	15.48606	−9.322	0.000***
Self-efficacy	30.6364	3.66804	36.9091	6.23626	−3.256	0.009**
Tenacity	26.1818	5.65364	37.2727	7.01557	−3.946	0.003**
Hope	28.9091	4.80530	34.1818	2.82199	−3.510	0.006**
Optimism	29.7273	3.84944	34.1818	2.82199	−4.750	0.001***
General well-being	68.7273	12.67352	78.7273	9.73746	−2.465	0.033*
Health concerns	10.2727	3.40855	9.1818	3.91965	0.643	0.535
Energy	14.7273	3.16515	20.2727	2.93567	−5.048	0.001***
Satisfaction and interest in life	5.3636	1.85864	6.4545	2.11488	−1.707	0.119
Melancholy or happy mood	13.2727	2.68667	16.2727	3.13340	−3.475	0.006**
Control of feelings and behavior	10.3636	1.36182	10.5455	1.29334	−0.296	0.774
Relaxation and tension	14.7273	4.02718	16.1818	3.02715	−1.066	0.311
Mental health	12.1818	4.44563	17.3636	1.74773	−2.053	0.001***
Self-affirmation	3.7273	2.90141	7.0000	1.26491	−4.297	0.001***
Melancholy	0.5455	0.93420	0.3636	0.92442	0.482	0.640
Anxiety	2.0000	1.84391	0.2727	0.64667	3.297	0.008**

Note: *p < 0.05; **p < 0.01; ***p < 0.001.

Significant improvements are observed in the scores of the experimental group members in both the overall psychological capital table and its individual dimensions after the implementation of multimedia system group counseling. This indicates a notable enhancement in individual psychological capital levels through the intervention of group counseling activities. Furthermore, there are noteworthy improvements in the scores of the general well-being questionnaire and general health questionnaire, underscoring a positive correlation between psychological capital, happiness, and mental health. As psychological capital levels increase, individual happiness and mental health also exhibit corresponding improvements. This aligns with Tom's assertion that “mental health is not merely the absence of mental illness but a continuous state of well-being.” In such a state, individuals can adeptly navigate their environment, exude vitality, and unleash their full potential. Moreover, the scores of the experimental group members undergo significant changes in the four dimensions of energy, depression or pleasant mood, self-affirmation, and anxiety both before and after the group counseling intervention. While dimensions such as health concerns, life satisfaction and interest, control of emotion and behavior, relaxation and tension, and depression do not show significant changes, their scores demonstrated varying degrees of improvement. This trend parallels the outcomes of Tom's research. Overall, elevating the level of psychological capital effectively contributes to heightened individual happiness and improved mental health.

## 5 Discussions

The outcomes pertaining to the variables of psychological capital, well-being, and mental health reveal the efficacy of the multimedia system group psychological intervention. Members in the experimental group exhibit higher levels of psychological capital, well-being, and mental health. Analysis of pre-test and post-test results for both the experimental and control groups prior to the multimedia system group psychological intervention reveal no significant disparities in psychological capital, well-being, and mental health. Following the implementation of multimedia intervention activities, a noteworthy improvement is observed in the psychological capital, well-being, and mental health of the experimental group members compared to their pre-intervention state. In contrast, the psychological capital, well-being, and mental health of the control group members remain unchanged. These findings underscore the significant positive impact of group counseling facilitate through a multimedia system on the psychological capital of college students. This study provides valuable insights for shaping future interventions and research initiatives within diverse educational contexts. To further enhance college students’ psychological capital, strategic measures are proposed, focusing on three pivotal aspects:

The augmentation of college students' psychological capital is pursued through multifaceted educational initiatives. A structured education system targeting the psychological capital of college students is meticulously developed. This includes the implementation of a dedicated psychological capital education course, delivering comprehensive knowledge systematically. Additionally, a diverse range of educational resources is leveraged to holistically foster students' psychological capital. This education is seamlessly integrated into implicit educational frameworks. Implicit education, characterized by tailoring activities to students' interests and creating immersive cultural atmospheres, serves as a subtle yet powerful method for guiding, developing, and educating students. The curriculum design employs instructional strategies that aim to stimulate, guide, and encourage students to actively engage in critical thinking, pose questions proactively, and showcase their inherent abilities. This approach nurtures students' interests and cultivates independent thinking and problem-solving skills, thereby enhancing their psychological capital. A scientific student learning evaluation system is formulated to provide clear life goals for college students. This system serves as a guiding framework, fostering the development and progress of students while creating a conducive environment for their overall growth. The implementation of a scientific learning evaluation system holds significant importance in elevating the psychological capital of college students. The symbiotic relationship between moral education and the cultivation of college students’ psychological capital is underscored. Moral education plays a pivotal role in shaping individuals with strong moral foundations, contributing substantively to the enhancement of psychological capital [31].

Efforts are dedicated to optimizing the cultivation environment for college students' psychological capital, with a focus on fostering a grateful campus culture. Recognizing the influential role of campus culture in shaping students' thoughts and behaviors, a positive impact on enhancing the hope and optimism of college students has been observed. The establishment of harmonious interpersonal relationships is prioritized as a pivotal element in cultivating students' positive psychological strength. Recognizing the vital role of interpersonal relationships, the emphasis is on fostering relationships that eliminate individual loneliness, provide substantial support during challenging times, facilitate quick recovery from predicaments, and contribute to overall happiness. The construction of a campus atmosphere conducive to harmonious interpersonal relationships guides students in effective communication, thereby constructively impacting the psychological capital of college students. Special attention is directed towards the mental and physical capital construction of students with dysfunctional family structures, particularly those from single-parent families. The school and educators are actively involved in providing ideological guidance, material support, and spiritual care. This holistic approach ensures that college students feel the warmth and support of the university community, contributing to elevated levels of optimism and hope. Different colleges and universities are encouraged to implement targeted measures tailored to the unique characteristics of their student body. These measures encompass diverse forms, rich content, and targeted strategies aimed at enhancing the psychological capital of college students. This approach acknowledges and addresses the diverse needs of students in various academic settings. The progressive development of students’ psychological capital is promoted across different academic grades. Tailoring strategies to the specific physiological and psychological characteristics of students at various stages, and considering the prevailing social environment and needs, ensures targeted and effective interventions for improving the psychological capital of college students [32].

A paramount focus is placed on reinforcing the self-improvement practices of college students' psychological capital. Central to this effort is the establishment of flexible life goals. Flexibility in life goals entails setting challenging objectives that stimulate individual enthusiasm, fostering a positive cycle of self-motivation. This approach is instrumental in promoting psychological capital by encouraging individuals to strive for and achieve their aspirations. Recognizing the inherent psychological phenomenon of interest in all individuals, emphasis is placed on discovering and nurturing potential self-interests. Interest serves as a powerful driving force, compelling individuals to seek knowledge and overcome challenges. By encouraging students to pursue achievements in their areas of interest, confidence is bolstered, contributing to the enhancement of individual psychological capital. The cultivation of innovative thinking is prioritized as a means to accelerate students' learning and living experiences. Fostering flexible thinking stimulates curiosity and propels students to actively seek knowledge. The challenges and joys encountered in problem-solving processes contribute significantly to tapping into students' positive psychological power. A deliberate effort is made to fortify the tenacious volitional quality among students. Willpower, defined as the psychological state where individuals ardently work toward their goals based on personal beliefs, is essential for navigating challenges. The process of developing indomitable will involves pursuing ideals and overcoming obstacles. Through this journey, individuals witness a gradual improvement in self-confidence, hope, optimism, and resilience. The cultivation of positive self-reflective awareness is actively encouraged. Regular self-reflection prompts individuals to objectively assess self-questions and accomplishments through introspection. This practice positively contributes to reinforcing students’ self-confidence and cultivating an optimistic outlook on life.

## 6 Conclusions

Based on empirical investigation and analysis, it is suggested that enhancing the psychological capital of college students can be achieved through the establishment and refinement of a psychological capital education system, optimization of the training environment for psychological capital, and reinforcement of self-improvement practices related to psychological capital. These proposals hold significant guiding and practical value. However, it is imperative to acknowledge the existing limitations such as an unstable theoretical foundation, sample concentration in surveys, and the need for refinement in the implementation of promotion strategies. These areas warrant further exploration in subsequent research. Drawing upon the study's findings, the following conclusion is evident: multimedia group psychological intervention activities yield positive and effective outcomes. While the impact on improving the hope dimension of psychological capital, well-being, and mental health may not be explicitly observed, these activities effectively enhance the well-being and mental health of participants in the experimental group by augmenting their psychological capital. The cultivation of college students' positive psychology and the improvement of their psychological capital prove beneficial in fostering physical and mental harmony, unlocking their potential, and bolstering their strengths. Consequently, these efforts positively influence various aspects of college students' lives, including their academic pursuits, personal well-being, and prospects in employment.

Several limitations exist within this study. Notably, the survey samples are confined in scope, and the generalizability of the survey results may be limited. The study primarily centers on the connotation of capital, its fundamental elements, and developmental aspects. Despite these shortcomings, the research holds substantial theoretical value and practical significance in the exploration of college students’ psychological capital. The innovative and challenging nature of the study underscores its potential contributions to the field. Future research endeavors should aim to address these limitations by delving into additional positive psychological factors.

## Data availability statement

All relevant data are within the paper and its Supplementary Material.

## CRediT authorship contribution statement

**Jinghui Zeng:** Writing – original draft, Data curation, Conceptualization. **Yangfen Chen:** Software, Resources, Methodology. **Yingying Zheng:** Writing – review & editing, Visualization, Validation, Supervision, Formal analysis.

## Declaration of competing interest

The authors declare that they have no known competing financial interests or personal relationships that could have appeared to influence the work reported in this paper.

## Figures and Tables

**Table:** Psychological capital questionnaire

Item	Totally agree	Relatively match	Unsure	Less sure	Strongly disagree
I believe I can analyze long-term problems and find solutions.	5	4	3	2	1
I believe that I can set my own goals for learning and career development.	5	4	3	2	1
I believe that I have the ability to achieve my own ideals.	5	4	3	2	1
I believe that I can effectively deal with unexpected things.	5	4	3	2	1
I am looking forward to the work and life I will start after graduation.	5	4	3	2	1
I am full of hope to achieve my goal in life.	5	4	3	2	1
At present, I am full of energy to pursue and complete my own learning and development goals.	5	4	3	2	1
If I find that my study or life is in trouble, I can think of many ways to get rid of or change the status quo.	5	4	3	2	1
In everyday life, when something is uncertain, I usually expect the best.	5	4	3	2	1
Most of the achievements I have made in my study and life are the result of my efforts and strong ability, not the result of good opportunities.	5	4	3	2	1
The setbacks and failures I have encountered in my study life are mostly due to my own insufficiency or roughness.	5	4	3	2	1
I firmly believe that “opportunities are only for those who are prepared.” I am relying on my own efforts to achieve my goals in life.	5	4	3	2	1
I think that if you want to graduate and find a good job, it is most important to choose a good tutor.	5	4	3	2	1
Generally, I can deal with the pressure and discomfort in study and life well.	5	4	3	2	1
Even in the process of learning, when I encounter tasks that I am not interested in but have to complete, I deal with them independently.	5	4	3	2	1
When the task is not going well, I can always think calmly and come up with a solution.	5	4	3	2	1
If I encounter setbacks and failures in my study and life, I can always recover quickly and move on.	5	4	3	2	1
When it comes to making decisions, I tend to do things my way.	5	4	3	2	1
I like to try my own ideas and try to understand and verify the feasibility of these ideas.	5	4	3	2	1
I like learning and working situations where I can do things in my own way.	5	4	3	2	1
I like the work that allows me to look at things from a new perspective.	5	4	3	2	1
When I encounter problems, I like to break the routine and try new problem-solving strategies and methods.	5	4	3	2	1
I always smile in the eyes of others.	5	4	3	2	1
The process of studying and participating in the research of the subject will make me anxious.	5	4	3	2	1
After completing the task, I will be immersed in the feeling of pleasure and happiness.	5	4	3	2	1
I love scientific research and learning new knowledge. I can easily devote myself to my study and work.	5	4	3	2	1
I hope that my mentor will arrange a novel, difficult task for me.	5	4	3	2	1
When I meet the problem that I am not sure to overcome, I will be very excited and happy.	5	4	3	2	1
I like to challenge problems that I'm not sure I can solve.	5	4	3	2	1
In order to pursue better performance, I can give up my leisure time to study and research.	5	4	3	2	1
The realization of my potential is the highest value goal I pursue.	5	4	3	2	1
My personal success is inseparable from the support and help of my family, teachers, classmates and friends.	5	4	3	2	1
I have been working hard all the time and hope that I can help others in the future.	5	4	3	2	1
During my student life, I gained more dignity, confidence and independence.	5	4	3	2	1
All the difficulties and setbacks I have experienced will be a valuable asset in my life, which will help me realize my personal value.	5	4	3	2	1
